# IL-27 enhances IL-15/IL-18-mediated activation of human natural killer cells

**DOI:** 10.1186/s40425-019-0652-7

**Published:** 2019-07-05

**Authors:** Yeon Ho Choi, Eun Jin Lim, Se Wha Kim, Yong Wha Moon, Kyung Soon Park, Hee-Jung An

**Affiliations:** 1 Institute for Clinical Research, CHA Bundang Medical Center, CHA University, Sungnam, Gyeonggi-do Republic of Korea; 2 Department of Pathology, CHA Bundang Medical Center, CHA University, Sungnam, Gyeonggi-do Republic of Korea; 3 Department of Medical Oncology, CHA Bundang Medical Center, CHA University, Sungnam, Gyeonggi-do Republic of Korea; 4 Department of Biomedical Science, CHA University, Sungnam, Gyeonggi-do Republic of Korea

**Keywords:** Natural killer (NK) cells, Natural killer cell receptor, Interleukin (IL)-15, Interleukin (IL)-27, Interferon-gamma

## Abstract

**Background:**

Natural killer (NK) cells are an emerging new tool for cancer immunotherapy. To develop NK cell therapeutics from peripheral blood mononuclear cells (PBMCs) of healthy donors, substantial expansion of primary NK cells is necessary because of the very low number of these cells in peripheral blood. In this study, we aimed to investigate the effect of various cytokine alone or combinations, in expanded NK cells and to analyze the synergetic effect of cytokine combinations.

**Methods:**

Human NK cells were isolated from healthy donor PBMC. Purified NK cells were stimulated with single cytokines or combinations of IL-2, IL-15, IL-18, and IL-27. The expanded NK cells were characterized by flow cytometry, cytotoxicity assay, calcein AM assay and Western blot.

**Results:**

We investigated the synergistic effects of each cytokine, namely, IL-2, IL-15, IL-18, and IL-27, on human NK cells isolated from PBMCs of healthy donors and cultured for 21 days. We identified that IL-15/IL-18/IL-27-mediated activation of NK cells most potently increased NK cell proliferation, cytotoxicity, and IFN-ɣ secretion compared with the activation observed with other treatments, including IL-2, IL-15, and IL-15/IL-18. Additionally, the expression of DNAM-1, NKG2D, CD69, and natural cytotoxicity receptors (NCRs; NKp30 and NKp44) increased on day 21 compared to that on day 0, demonstrating the activation of NK cells. In vitro, expanded NK cells were highly cytotoxic against cancer cells, displaying increased perforin and granzyme B accumulation.

**Conclusions:**

Taken together, these results indicated that IL-27 can synergize on NK cell expansion and activation with IL-15 and IL-18. In addition, we described an improved culture method for ex vivo expansion of human NK cells with IL-15/IL-18/IL-27 stimulation and characterized the response of NK cells to this stimulation.

**Electronic supplementary material:**

The online version of this article (10.1186/s40425-019-0652-7) contains supplementary material, which is available to authorized users.

## Background

In humans, natural killer (NK) cells constitute approximately 10–15% of lymphocytes and are typically defined as CD3^−^CD56^+^ cells [1]. NK cells are effectors of the innate immune system involved in the clearance of tumor cells and virus-infected cells [2–5]. They mediate innate immunity through direct cell lysis by intracytoplasmic azurophilic granules [6, 7] and modulation of other immune cells by release of proinflammatory cytokines, such as interferon (IFN)-ɣ and tumor necrosis factor (TNF)-ɑ [1]. The effector function of NK cells is regulated by the balance between activating and inhibitory receptor signals [8]. NK cell activation is regulated by a variety of surface receptors, such as the CD16/FcRIII receptor, natural cytotoxicity receptors (NCRs; NKp46, NKp44, and NKp30), natural killer group 2D (NKG2D), 2B4, and costimulatory receptors (DNAM-1) [9]. In contrast, NK cell inhibition is regulated by killer cell immunoglobulin-like receptors (KIRs) and CD94/NKG2A, which recognize major histocompatibility complex (MHC) class I molecules on target cells [8]. Therefore, MHC class I-deficient cancer or transformed cells are highly sensitive to NK cells. Because of these inherent functions of NK cells, they have attracted attention as promising immunotherapeutics for cancer and viral infections [10].

Human NK cells may be divided into two subsets, CD56^dim^ and CD56^bright^ [2]. In peripheral blood, the major subpopulation is CD56^dim^CD16^bright^ (≥ 90% of total NK cells, primarily CD56^dim^) and is commonly described as the most cytotoxic subset, whereas CD56^bright^CD16^dim/−^ NK cells (5–10%, primarily CD56^bright^) are abundant cytokine producers [11, 12]. However, NK cell heterogeneity is still poorly characterized, even though NK cells play an essential role in target-cell killing and cytokine secretion.

In general, NK cells are well known for their ability to be maintained alive in long-term culture as well as for their ability to be activated when treated with different types of cytokines [13, 14]. The role of cytokines in controlling NK cell responses has been an area of intense research. The gamma receptor (γcR)-interacting cytokines, interleukin (IL)-2, IL-15, and IL-21 or their combinations have been used to expand NK cells [14–18]. Traditionally, IL-2 has been extensively used to expand murine and human NK cells [19, 20]. IL-15 is a gamma-chain signaling cytokine that plays a critical role in NK cell differentiation and survival [21, 22]. It activates the PI(3)K-mediated mTORC1 pathway [23]. IL-2 and IL-15 are the best-studied cytokine activators of NK cells and have many positive functional effects on NK cells to improve antitumor responses [24, 25]. In NK cells, IL-18 has been usually described as a costimulatory cytokine that functions synergistically with IL-12 and IL-15 [26, 27]. IL-18 is a member of the IL-1 family that interacts with a heterodimeric receptor composed of IL-18Rα and IL-18Rβ [28]. Additionally, IL-18 enhances NK cell effector functions, including IFN-γ secretion [17]. IL-27 is a heterodimeric cytokine that belongs to the IL-12 family and consists of p28 and Epstein–Barr virus-induced gene 3 (EBI3), signaling through the IL-27R composed of WSX-1 and CD130/gp130 [29, 30]. IL-27 modulates the antitumor cytotoxic response of NK cells [31–34]. However, the cellular mechanisms underlying IL-27-mediated immune regulation remain unclear.

In this study, we investigated the ability of each cytokine and their combination on expansion and activation of primary NK cells. We presented data showing that IL-15, IL-18, and IL-27 potently enhance NK cell cytotoxicity and increase the absolute number of CD3-CD56+ NK cells in long-term culture (21 days). Our results suggest that synergistic interactions between IL-15, IL-18, and IL-27 play a crucial role in CD3-CD56+ NK cell functions by improving NK cell cytotoxicity activity, which increases perforin granule accumulation and IFN-γ production. We further characterized the phenotypic and functional consequences of CD3-CD56+ NK cells being stimulated with IL-15, IL-18, and IL-27.

## Methods

### Human natural killer (NK) cell isolation and ex vivo expansion

Primary NK cells were purified (> 95% pure) from peripheral blood mononuclear cells (PBMCs) of healthy human volunteers (*n* = 26; female *n* = 12, male *n* = 14). This research protocol was reviewed and approved by the institutional review board of CHA Bundang Medical Center, CHA University (permit number: CHAMC 2017–01–001). PBMCs were isolated from whole blood by a density gradient with Ficoll-Paque Plus (GE Health Care, Piscataway, NJ) followed by purification using an NK cell isolation kit (Miltenyi Biotec, Germany). Cells were activated in CellGenix® GMP Stem Cell Growth Medium (SCGM, CellGenix, Freiburg, Germany) supplemented with 10% human serum (Sigma-Aldrich, St. Louis, MO) and the cytokines IL-2 (10 or 100 ng/ml), IL-15 (10 ng/ml), IL-27 (10 ng/ml) (Peperotech, Inc. NJ) and IL-18 (10 ng/ml) (R&D Systems, Inc., MN) under standard culture conditions (a humidified 5% CO_2_ atmosphere, 37 °C) for 21 days (long-term culture). Fresh culture medium containing cytokines and 10% human serum was added to the flask every 2 to 3 days for 21 days. The initial seeding density of NK cells was a median of 1 × 10^6^ (range: 0.8–1.3 × 10^6^) NK cells in 6-well plates (SPL Life Sciences, KOR). After 7 days, the cells were transferred into T25 flasks, T75 flasks, and T175 flasks (SPL Life Sciences, KOR) with additional media containing cytokines for 21 days.

### Morphological study

Morphological appearances of NK cells were observed after 7 days for short-term, 14 days for mid-term, and 21 days for long-term cultures using phase contrast microscopy (Nikon, Tokyo, Japan). Cells were stained with May-Grüwald-Giemsa solutions. Images were analyzed using an ICC50 HD Camera System from Leica (Leica Microsystems).

### Human cell lines

The human tumor cell line K562 (human chronic myelogenous leukemia, CML) and A2780 (human ovarian cancer cell line) were obtained from the ATCC. Cells were cultured in RPMI 1640 medium (Gibco/Life Technologies, Carlsbad, CA) supplemented with 10% heat-inactivated FBS, 200 mM L-glutamine and 10,000 U/mL Pen/Strep (Gibco/Life Technologies, Carlsbad, CA) at 37 °C in a 5% CO_2_ incubator. NK-92, the human NK cell line, was purchased from the ATCC and cultured in Alpha MEM devoid of ribonucleosides and or deoxyribonucleosides, 12.5% FBS, 10,000 U/mL Pen/Strep (Gibco/Life Technologies, Carlsbad, CA), 2 mM L-glutamine (Gibco/Life Technologies, Carlsbad, CA), 0.2 mM inositol (Sigma-Aldrich, St. Louis, MO), 0.02 mM folic acid (Sigma-Aldrich, St. Louis, MO), 0.1 mM 2-mercaptoethanol (Sigma-Aldrich, St. Louis, MO), and 100–200 U/ml recombinant IL-2 (Peperotech, Inc., NJ).

### Phenotypic analysis of expanded NK cells

Day 21-expanded NK cells as well as freshly isolated PBMCs and NK-92 cells, were analyzed for phenotypic markers by flow cytometry. Cells were stained in Fluorescence-activated cell sorting (FACS) buffer on ice for 30 min with mixed antibodies. The following fluorescently labeled antibodies were purchased from BD Pharmingen™: anti-human CD3 (HIT3a), CD56 (B159), CD16 (3G8), CD314 (NKG2D; 1D11), CD335 (NKp46; 9E2), CD336 (NKp44; p44–8), CD337 (NKp30; p30–15), CD96 (6F9), CD226 (DNAM-1; DX11), CD19 (HIB19), CD14 (M5E2), KIR2DL1/CD158a (HP-3EA), KIR2DL2/3/CD158b (CH-L), and KIR3DL1/CD158e1 (DX9). The antibodies for human NKG2A/CD159a (#131411), KIR2DL4/CD159d (#181703), KIR3DL2/CD159k (#539304), and KIR3DL3/CD158z (#1136B) were purchased from R&D systems. The antibodies for human CD69 (CH/4) were purchased from Invitrogen, and antibodies for human KIR2DL5A/CD158f (UP-R1) were purchased from Origene. After washing with FACS buffer, cells were analyzed on a FACS Calibur machine (BD Biosciences). Data analysis was carried out using the FlowJo software program (Tree Star Inc., San Carlos, CA).

### Enzyme-linked immunosorbent assay (ELISA)

At each time point (7, 14 and 21 days), 500 μl of cell supernatants were collected and frozen at − 80 °C. Cytokine analysis was performed according to the manufacturer’s instructions using a Human IFN-ɣ Quantikine ELISA Kit (R&D Systems, Minneapolis, MN). Absorbance was read at 450/540 nm using a SpectraMax L Microplate Reader (Molecular Devices, Sunnyvale, CA). Concentration was calculated using the standard provided with the kits. Means and standard deviations of concentrations in triplicate samples were compared by *t*-test.

### Calcein AM release assay

Calcein (calcein AM, AM; acetoxymethyl) is a fluorescent dye that can be used to determine cell viability in eukaryotic cells (C3099, Thermo Fisher Scientific). K562 target cells were used to demonstrate the NK cell-mediated cytotoxicity detection method using calcein AM. Target cells were stained by incubating them in 5 μM calcein AM staining media (1 mg/ ml stock) in complete RPMI media for 30 min at 37 °C in 5% CO_2_. K562 tumor targets (T, loaded with calcein) and primary NK cells (E, effector cells) stimulated with IL-15/IL-18/IL-27 for 21 days were seeded together in a cell culture slide (8 chambered, SPL Life Sciences, Korea) at different E:T ratios (10:1, 5:1, 2.5:1, 1.25:1 and 0:1) in duplicate. Cells were cocultured at 37 °C for 4 h. After 4 h, live imaging of NK cell cytotoxicity on tumor targets was performed using a Zeiss LSM 510 microscope (Carl Zeiss Microscopy, LLC). The cytotoxicity % was calculated by the equation: cytotoxicity % = (dead target cell count / dead + live target cell count) × 100.

### Immunocytofluorescence staining

Cells were washed with cold PBS, and 200 μl of the cell suspension (1 × 10^4^ cells/ml) was added to a slide funnel and fixed in a cold methanol-acetone solution for 5 min. The slides were washed three times with PBS and then blocked with blocking solution (10% normal goat serum, Jackson ImmunoResearch Laboratories Inc., West Grove, PA) for 1 h. The cells were then washed with PBS and permeabilized in 0.1% Triton® X-100 (in PBS) for 20 min. Protein was detected using anti-perforin (1:100, Abcam) and anti-granzyme B (1:100, Abcam) antibodies for 24 h at 4 °C, followed by several washes in PBS. Incubation was repeated with an appropriate fluorescein isothiocyanate and Texas Red-conjugated secondary antibody (1:100, Jackson ImmunoResearch Laboratories Inc., West Grove, PA), and cell nuclei were stained with 4,6-diamidino-2-phenylindole. Coverslips were mounted onto slides with Vectashield Mounting Medium (Vector Laboratories, Inc., Burlingame, CA) and examined using a Zeiss LSM 510 confocal microscope (Carl Zeiss, Inc.).

### Western blot analysis

Cells were lysed in ice-cold RIPA buffer (#9806, Cell Signaling Technology, Inc. Danvers, MA). Before isolation of total protein, NK cells were gently removed  by washing with 1 X cold PBS.  For Western blotting, anti-caspase-3, anti-caspase-8, and anti-caspase-9 antibodies from Cell Signaling; anti-perforin (CB5.4) and anti-granzyme B antibodies from Abcam; and anti-β actin antibodies from Santa Cruz Biotechnology were used. Secondary antibodies and ECL reagents were obtained from GE Healthcare. Signals were visualized by a G: BOX-CHEMI-XT4 Gel Documentation System.

### Luminescent cytotoxicity assay

The cytotoxicity of NK cells was determined using a CytoTox Glo™ Cytotoxicity Assay (Promega Corporation, Madison, WI) according to the manufacturer’s instructions. Briefly, K562 cells were seeded at a density of 5.0 × 10^4^ cells per well in 96-well plates (SPL Life Science, KOR). Then, 1.0 × 10^6^ expanded NK cells and NK-92 cells were resuspended in SCGM complete medium, and four serial dilutions (2-fold) were performed. Aliquots from each NK cell serial dilution containing 5.0 × 10^5^, 2.5 × 10^5^, 12.5 × 10^5^ and 6.25 × 10^4^ cells were added per well in a 96-well plate in duplicate. After 4 h of incubation, 50 μl of CytoTox Glo™ Cytotoxicity Assay reagent was added to all wells. This assay used a luminogenic peptide substrate to detect dead cells by selectively measuring ‘dead-cell protease activity.’ The luminescent signal that reflects cytotoxicity was measured using a SpectraMax L Microplate Reader (Molecular Devices, Sunnyvale, CA). Cytotoxicity was calculated by dividing the luminescent dead-cell signal by the total cell luminescence value.

### Statistical analysis

A two-tailed paired t-test was performed to analyze data using GraphPad Prism 5.0 software (GraphPad Software Inc., San Diego, CA) as indicated in the figure legends. Statistical analysis was performed using a t-test adjusted with Benjamini and Hochberg procedure, and the ANOVA test adjusted with Bonferroni posttests. *P* values of less than 0.05 were considered statistically significant. The results are expressed as the mean ± SD and were obtained from two or three independent experiments.

## Results

### Characterization of PBMCs and NK cells in healthy donors

In this study, we evaluated the characterization of NK cells derived from PBMCs from 7-, 14-, and 21-day cultures. First, we isolated peripheral blood NK cells from healthy donors (HDs). Twenty-six healthy adult donors enrolled in the study. Twelve donors were females with a mean age of 33.25 ± 5.429 years (*n* = 12, mean ± SD); the range was 20–40 years. Fourteen donors were males with a mean age of 35.07 ± 5.313 years (*n* = 14, mean ± SD); the range was 20–49 years (Additional file 1: Table S1).

In peripheral blood, human NK cells comprise approximately 10–15% of the total cells. The results from female donors show that the mean total viable PBMC count at presentation was 2.080 ± 0.871 × 10^8^ / 100 ml (mean ± SD, range: 1.070–4.0 × 10^8^ / 100 ml), including 9.34% (range: 5.374–13.931%) CD3-CD56^dim^ NK cells (Additional file 2: Figure S1). The mean total viable PBMC count from male donors was 1.869 ± 0.617 × 10^8^ / 100 ml (mean ± SD, range: 1.02–2.64 × 10^8^ /100 ml), including 12.62% (range: 6.786–23.137%) CD3-CD56^dim^ NK cells. Our data show that the NK cell populations did not significantly differ between the age groups of males and females. After purification of NK cells, we obtained 93.68 ± 4.30% CD3-CD56^dim^ NK cells. On day 21, NK cells stimulated with single cytokines or a combination were composed of highly enriched CD3 − CD56^dim^ (96.34 ± 2.41%) or CD56^dim^ CD16+ (93.10 ± 3.38%) NK cells (Additional file 2: Figure S2), with minimal contamination by CD3+ T cells (0.11 ± 0.21%), CD14+ monocytes (0.63 ± 0.32%) or CD19+ B cells (0.79 ± 0.54%). 

### Effect of IL-27 on human NK cell proliferation and cytotoxicity

To improve the ex vivo proliferation of human NK cells, we optimized the culture medium using different types of cytokines, such as IL-2, IL-15, IL-18, IL-27, and cytokine combinations. To test this approach, we purified CD3-CD56^+^ NK cells from PBMCs (median purity: 90.8%, range: 93.1–97.5%).

**Fig. 1:**
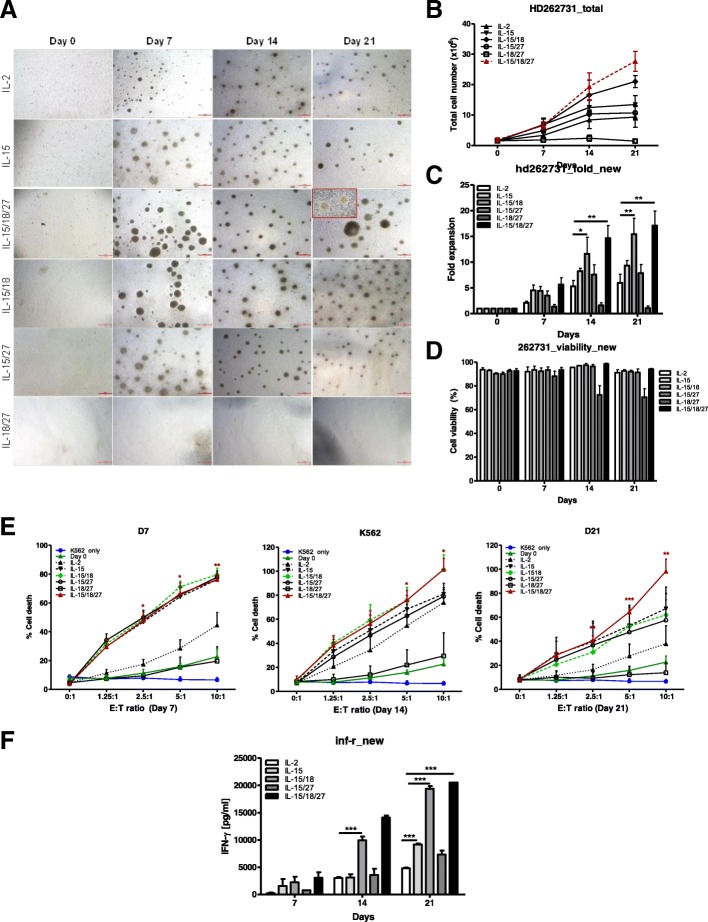
Cytokine regulation of the proliferation and cytotoxicity of primary NK cells*.* **a** CD3-CD56+ NK cells (0.6–1.8 × 10^6^ / well) isolated from PBMCs were cultured in the indicated cytokines for 21 days. Primary NK cells were imaged using an inverted microscope and counted. Representative images of aggregates of growing primary NK cells in different culture conditions. Bars represent 500 μm; original magnification × 40. **b** The graph represents the total NK cell number of each group. Symbols indicate cytokine treatment groups (*n* = 3 / group): IL-2 (▲), IL-15 (▼), IL-15/18 (◆), IL-15/27 (○), IL-18/27 (□), and IL-15/18/27 (*red*). **c**. Fold expansion of NK cell numbers compared with those on day 0 following culture of CD3-CD56+ NK cells with the indicated cytokines. The graphs show the mean ± SD. * *P* < 0.05, ** *P* < 0.01, and *** *P* < 0.001, compared with day 0. **d** NK cell viability. The viable cell numbers were determined by trypan blue staining on days 7, 14 and 21. * *P* < 0.05, compared with day 14. **e** NK cytotoxicity assays of various cytokine-stimulated NK cells with K562 target cells on days 7, 14 and 21. The E:T ratios ranged from 0:1 to 10:1. After 4 h of incubation at 37 °C, the lysis of target cells was measured by ELISA. E:T indicates the effector-to-target ratio. The cytolytic activity of human NK cells stimulated with IL-15/18/27 toward K562 cells was significantly increased (*P < 0.05, **P < 0.01, *** *P* < 0.001) compared with that of resting NK cells (day 0) at the same E:T ratio. **f** Supernatants were analyzed for IFN-ɣ secretion by ELISA. The data presented are the mean ± SD of three separate experiments., *** *P* < 0.001, compared with IL-2 treated group

Purified NK cells were cultured with IL-2, IL-15 or cytokine combinations, such as IL-15/IL-18, IL-15/IL-27, IL-18/IL-27, and IL-15/IL-18/IL-27, for 7 days for short-term, 14 days for mid-term, and 21 days for long-term cultures (Fig. 1a).

Interestingly, NK cell proliferation was not seen for stimulation with IL-18 only, IL-27 only (data not shown) or IL-18/IL-27. After 7 days of culture, the proliferation of NK cells was markedly decreased with IL-18 or IL-27 only treatment. The total cell number at day 14 was ~ 50% of that on day 0. In particular, the growth stopped in two samples with IL-18-only or IL-27-only cultures on day 14, and the cell number subsequently decreased. Additionally, NK cell clusters gradually decreased after 7 days. We obtained similar results with a combination of IL-18 and IL-27. In NK cells, IL-18 or IL-27 has usually been described as a costimulatory cytokine that functions synergistically with IL-15 [27, 33].

Many cytokines have been reported to enhance the proliferation of NK cells alone or synergistically with other stimuli [35]. In this study, after 21 days of culture, a combination of IL-15/IL-18/IL-27 showed the highest NK cell expansion, followed by IL-15/IL-18 and IL-15, leading to a total median number of 34.8 × 10^6^ (range: 23.6–41.9 × 10^6^) viable NK cells (Fig. 1b), corresponding to a median fold-expansion of 17.19 ± 4.85-fold with 94.15 ± 1.05% viability (Fig. 1c-d). We also measured the cytotoxic activity of the stimulated NK cells with different combinations of cytokines (Fig. 1e). The cytotoxic activity of NK cells derived from different NK culture batches was analyzed in different effector/target ratios (0:1, 1.25:1, 2.5:1, 5:1 and 10:1) against target K562 cells and compared with the basal cytotoxicity of resting NK cells (day 0). After 14 days of culture, NK cells stimulated with IL-15/IL-18/IL-27 showed significantly increased cytotoxicity in response to contact with K562 target cells compared to that for other cytokine combinations. We obtained similar results using A2780 cells as the target cells (Additional file 2: Figure S3). Additionally, the expansion of CD3-CD56+ NK cells incubated with IL-15/IL-18/IL-27 resulted in a significantly increased production of IFN-ɣ (Fig. 1f) at days 14 and 21. These results suggest that IL-27 acts synergistically with IL-15 and IL-18 in promoting NK cell cytotoxicity.

### Comparison of NK cell receptor expression in expanded NK cells

**Fig. 2:**
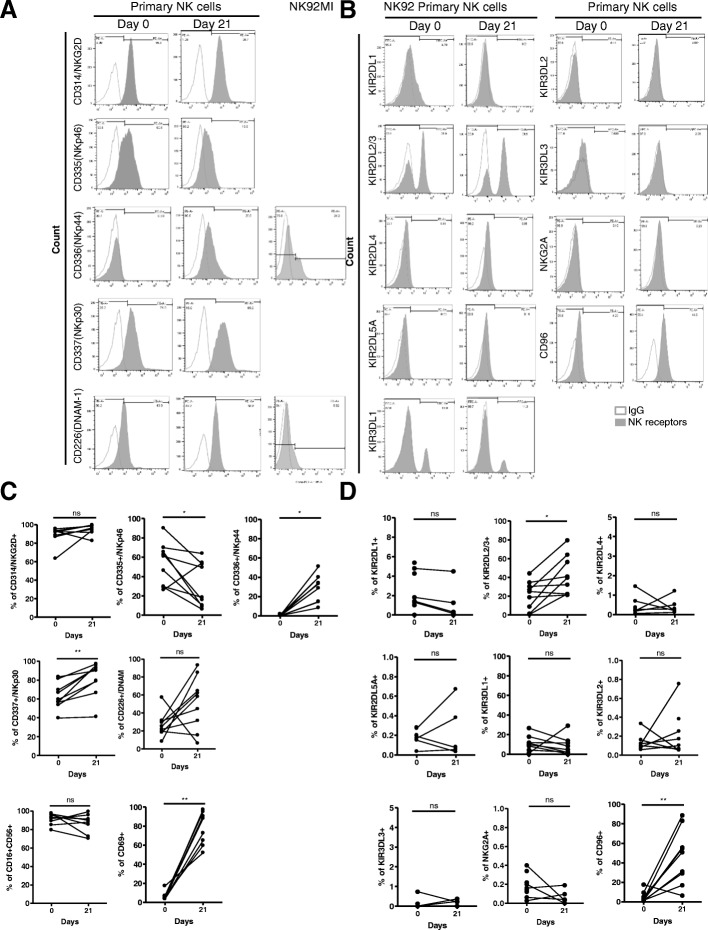
Flow cytometry analysis of NK cell receptors on human primary NK cells. (A) Cell surface expression of the indicated molecules on primary human NK cells on day 0 and day 21. Cells were stimulated with cytokine combinations (IL-15, IL-18, and IL-27) for 21 days. Primary NK cells from healthy donors were stained for expression of NK cell activating receptors (**a**) and inhibitory receptors (**b**), as indicated. Histograms show representative examples of NK cell receptor expression (shadow area) and show the percentage of NK cells positive for a given receptor relative to the isotype control (gray lines). NK cells were gated as viable, single, CD3-CD56+ cells. (**c**-**d**) Statistical analysis for the difference in NK cell receptor expression between day 0 and day 21. Significant differences are indicated in the graph as follows: *P < 0.05 and ** *P* < 0.01, compared with day 0. n.s: not significant

We examined the effect of combined treatment with IL-15, IL-18, and IL-27 on the expression of NK cell receptors. We investigated the expression pattern of the NK cell activating receptors CD16, NKG2D, and CD69, the natural cytotoxicity receptor (NCR) family, such as NKp44, NKp30, NKp46, DNAM-1, and CD69 (Fig. 2a), and inhibitory receptors [killer-cell immunoglobulin-like receptors (KIRs), such as KIR2DL1, KIR2DL2/3, KIR2DL4, KIR2DL5A, KIR3DL1/2/3, CD94/NKG2A and CD96] (Fig. 2b) on NK cells before and after cytokine-mediated NK cell expansion using flow cytometry and compared these values to those of NK-92 and NK-92MI cell lines (Additional file 2: Figure S4). As shown in Fig. 2a, after 21 days of culture with IL-15/IL-18/IL-27, the expression levels of most NCR family members, such as NKG2D, NKp44, NKp30, and CD69, were increased compared to those in resting NK cells. The expression of one of the coreceptors, CD226, was also increased after culture for 21 days. However, CD335 (NKp46) expression was decreased (Fig. 2c). The expression level of the most inhibitory receptors (Fig. 2d) did not change during cytokine treatment, except for the expression of KIR2DL2/3 and CD96, which was increased in expanded NK cells [KIR2DL2/3 (day 0: 20.75 ± 16.01; day 21: 44.60 ± 20.54) and CD96 (day 0: 4.16 ± 5.57; day 21: 47.63 ± 28.07)] (Additional file 1: Table S2). We also observed IL-2^Hi^, IL-2 alone, and IL-15 alone groups (Additional file 1: Table S3). The expression of one of the NCRs, NKp30, was increased in the IL-2- and IL-15-only stimulated groups. Interestingly, KIR2DL2/3 expression was observed only in the stimulated IL-2^Hi^ or IL-2-only groups.

We also measured programmed death-1 (PD-1) and cytotoxic T lymphocyte-associated antigen 4 (CTLA-4) expression. The expression levels of PD-1 and CTLA-4 did not significantly change after 21 days of culture, similar to NKG2A (day 0: 0.17 ± 0.11; day 21: 1.12 ± 1.51) (Additional file 1: Table S3).

### IL-27 acts synergistically with IL-15 and IL-18 to increase perforin accumulation in expanded NK cells

**Fig. 3:**
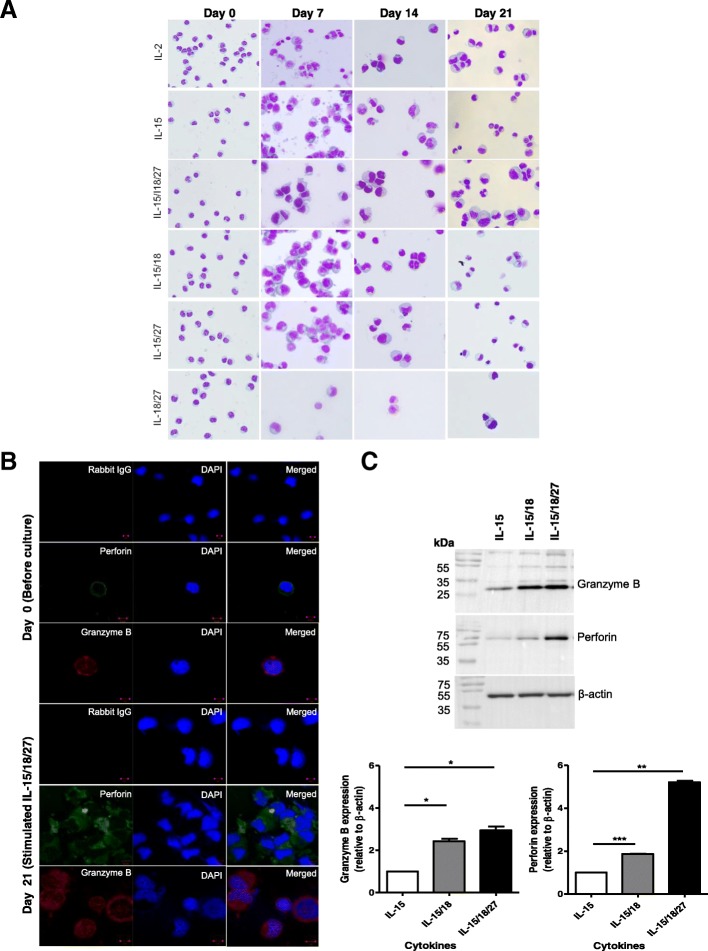
Increased cytolytic granule accumulation in expanded NK cells. **a** The morphology of expanded NK cells. Cytospin preparations of NK cells (day 0, resting NK cells) and cytokine-activated NK cells (day 21) were stained by Wright-Giemsa staining. Representative images from cultures at 7, 14, and 21 days are shown. Original magnification × 400. **b** Immunofluorescence staining for perforin and granzyme B for NK cells before (day 0, *upper panel*) and after culture (day 21, *lower panel*) in the presence of a cytokine combination (IL-15/IL-18/IL-27). DAPI was used to stain the nuclei (blue). The negative control using secondary antibody (anti-IgG) only demonstrated low nonspecific binding of the secondary antibody. The data shown are representative of three independent experiments. Bars represent 10 μm. Original magnification × 100. **c** Western blotting analysis for perforin and granzyme B. NK cells were stimulated with cytokines (IL-15, IL-15/IL-18, and IL-15/IL-18/IL-27) for 21 days. The graph represents the relative expression of each protein. Protein bands were quantitated by densitometric analysis. The ratio of the intensity of protein bands relative to that of β-actin was calculated. Experiments were repeated three times with similar results. *P < 0.05, compared with cells cultured in the presence of IL-15 alone

To investigate whether the morphological changes were the result of cytokine stimulation after 7, 14 and 21 days, we performed Wright-Giemsa staining. As shown in Fig. 3a, Giemsa staining revealed that IL-2, IL-15, IL-15/IL-18, IL-15/IL-27, and IL-15/IL-18/IL-27 affected morphological changes in CD3-CD56+ NK cells during culture, including increased size and cytoplasmic granularity. With the assumption that these cytoplasmic granules are cytolytic agents of NK cells, such as perforin and granzyme B, we analyzed the subcellular locations of these proteins using immunofluorescence staining (Fig. 3b).

Our data show that perforin and granzyme B signals were evenly distributed and compactly stained in the enlarged cytoplasm of 21-day IL-15/IL-18/IL-27-expanded NK cells (*lower panel*), which were more abundant than those of resting NK cells (*upper panel*). Using Western blots, we then compared the expression levels of these proteins in NK cells incubated with IL-2^hi^, IL-2^low^, IL-15, IL-15/IL-18, IL-15/27 or IL-15/IL-18/IL-27 (Fig. 3c, Additional file 2: Figure S5). The expression of granzyme B and perforin in NK cells cultured with IL-15/IL-18/IL-27 was significantly increased compared with that in cells cultured with IL-15 alone (2.94-fold, granzyme B; and 5.2-fold, perforin) or IL-15/IL-18 (1.2-fold, granzyme B; and 2.82-fold, perforin, Additional file 2: Figure S6).

These data suggest that IL-27 plays an essential role in combination with IL-15 and IL-18 in expanding the CD3-CD56+ population and contributes to CD3-CD56+ NK cell cytotoxic activation by increasing cytolytic granule accumulation, especially perforin.

### IL-27-treated NK cells display augmented caspase-dependent apoptotic activity in target cells

**Fig. 4:**
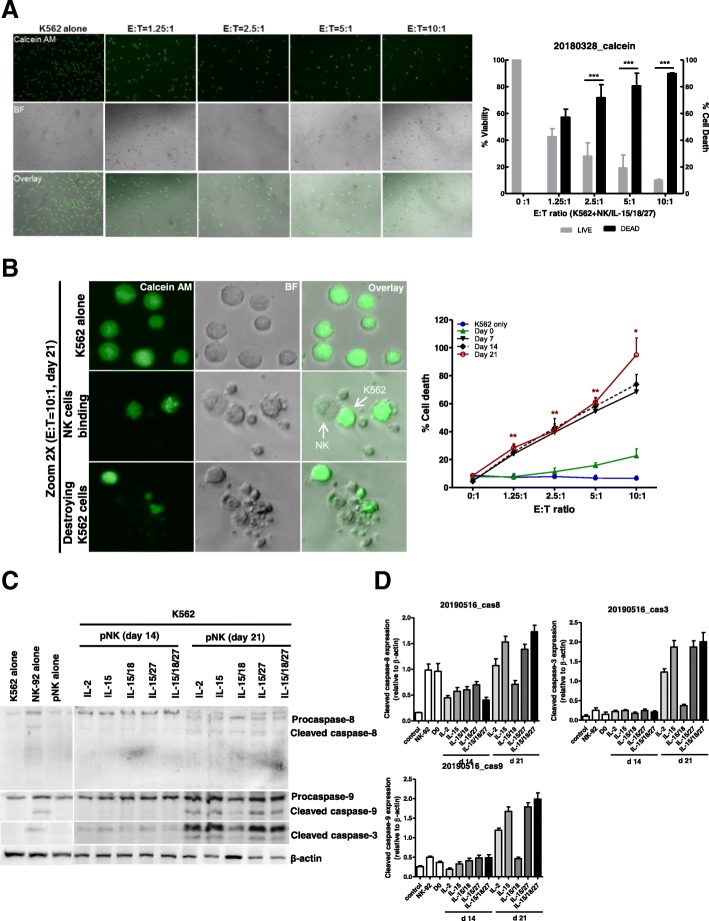
Measurement of NK cell cytotoxicity by imaging cytometry. **a** K562 target cells were stained with calcein AM. After 4 h of incubation with 21-day-expanded NK cells (stimulated with IL-15/18/27), fluorescence images show progressive loss of fluorescence intensity of the K562 cells at various E:T ratios. Representative bright field, calcein, and overlay images showing E:T ratio-dependent target cell killing. Original magnification × 100. The graph represents the percentage of viable or dead target cells. *** *P* < 0.001 was considered signigicant. **b** A high power view of the calcein AM assay showing the progress of NK cell killing. Nearly all of the target cells were killed in a 10:1 effector-to-target cell sample, while calcein AM-labeled K562 cells were not killed in the control image. Bright-field and fluorescence overlay images of calcein show K562 cells undergoing apoptotic death following interaction with NK cells. The images were derived from a Zeiss LSM 510 microscope (*left*). The graph (*right*) represents cytotoxicity against K562 cells with expanded NK cells on days 7, 14 and 21 over the 4 h of the assay. *P < 0.05, ** *P* < 0.01, ***P <0.001 compared with day 0. Symbols indicate cytokine treatment groups (*n* = 3 / group): day 0 (▲), day 7 (▼), day 14 (◆), day 21 (red ○), and K562 cells only (●). **c** Immunoblot analysis for caspase-8, − 9 and − 3 activation. K562 cells were cocultured with primary NK cells for 4 h. Immunoblotting was performed with antibodies specific for caspase-8, − 9 and − 3 and their cleaved forms. β-actin was used as an internal standard. **d** Protein bands were quantitated by densitometric analysis. The ratio of the intensity of protein bands relative to that of β-actin was calculated. Bar graph represents the relative expression of cleaved caspase-8, − 9 and − 3 proteins. Experiments were repeated three times with similar results

Next, we investigated whether expanded NK cells could directly kill cancer cells, using a chamber slide as a coculture system. After 4 h of coculture with K562 and expanded NK cells, we performed a calcein release assay. Figure 4a shows representative bright-field (*middle panel*) and fluorescence images (*upper panel*) at each E: T ratio for the target cells. The number of brightly fluorescent live target cells gradually decreased with increasing number of expanded NK cells (E: T ratio), while all of the control target cells (K562 alone) were brightly fluorescent. At an E: T ratio of 5:1, nearly total lysis of K562 cells was observed. To derive live cell counts, the fluorescence intensity of the target cells was assessed at different E: T ratios so that target cells with lower fluorescence signals, such as those lacking calcein release and apoptotic bodies, were excluded from the live (bright) cell counts (Fig. 4a, b). In Fig. 4b, we demonstrated that K562 cells showed dramatic morphological changes typical of apoptotic death, such as blebbing and cell shrinkage, after 4 h of coculture with expanded NK cells.

Finally, we investigated the influence of various cytokine combinations on CD3-CD56+ NK cell cytotoxicity by measuring caspase activity in K562 and A2780 cells (Additional file 2: Figure S3). We tested this effect because NK cells are generally known to rapidly eliminate target cells by two different pathways, including the death receptor pathway and the granule-dependent pathway [36]. As shown in Fig. 4c and d, killing of K562 cells by 21-day-expanded NK cells was associated with caspase-3, − 8, and − 9 activations and this effect was observed for NK cells expanded with IL-15, IL-15/IL-27, and IL-15/IL-18/IL-27. By contrast, cleaved forms of these caspases were barely detected in the day 14 According to our data, after 21 days of culture with IL-15, IL-18, and IL-27 stimulated NK cells to recover caspase activation on target cells and enhance the activity of NK cells to target cells. On the other hand, 7, and 14-day-expanded NK cells did not induce caspase pathway activation in the K562 and A2780 cell lines (Additional file 2: Figure S3). These results indicate that caspase-dependent apoptosis is one of the mechanisms by which NK cells mediate killing and that IL-27 may be crucial to the activation of this process in human NK cells.

## Discussion

Many researchers have recently tried to use cell therapy to eliminate tumors from cancer patients. From an immunotherapeutic approach, NK cells are considered to be a promising effector immune cell type for new cancer therapy. Although many kinds of autologous and allogeneic NK cells have been known to mediate antigen-independent tumor cytotoxicity activity [3], the therapeutic potential of NK cell-based immunotherapy is still unclear. PBMCs have been used as one of the sources of NK cells for clinical applications. However, their clinical application has been limited because it is challenging to prepare sufficient numbers of NK cells. Thus, ex vivo NK cell expansion is the most critical step in developing NK cell therapy.

Prior studies have used various NK cell expansion protocols to develop NK cell therapeutics [37]. The use of feeder cells has been an essential method for securing many cells that can have a therapeutic effect. However, the use of feeder cells might induce the problem of infectious disease and the regulatory hurdles in the manufacturing pharmaceutical products, although it had an advantageous for NK cell expansion. We, therefore, sought to find a simplified method on NK cell proliferation and activation using a new cytokine combination without feeder cells.

In the present study, we found that human NK cell expansion and activation stimulated by IL-15/IL-18/IL-27 were significantly more than those stimulated by other combinations of cytokines, including IL-15/IL-18, IL-2 alone, or IL-15 alone, which were performed in prior studies [38]. Based on our results, it seems reasonable to combine the different cytokines for ex vivo stimulation of human CD3-CD56+ NK cells, although not many reports have claimed that NK cells can be expanded from PBMCs [39, 40].

Human NK cell expansion requires multiple signals for survival, proliferation, and activation. Human NK cell cultures traditionally are maintained for 14 to 28 days and require frequent manipulations, such as media changes, to refresh cytokines and other growth factors and cell splitting to ensure that NK cells are maintained at a concentration that optimizes their growth and viability [41]. In this study, we demonstrated that several cytokines, such as IL-2, IL-15, IL-15/IL-18, and IL-15/IL-18/IL-27, were able to induce CD3-CD56+ NK cell proliferation. However, IL-18/IL-27 without IL-15 did not induce CD3-CD56+ NK cell proliferation (Fig. 1b), suggesting that IL-18 and IL-27 alone are not enough for CD3-CD56+ NK cell proliferation. This finding is consistent with a previous report in which IL-15-deficient mice lacked mature NK cells, supporting the idea that IL-15 is required for NK cell survival [21].

IL-27 belongs to a family of cytokines that includes IL-12, IL-23, and IL-35 [29, 42]. IL-27 directly triggers IFN-ɣ secretion without affecting the production of other cytokines, such as IL-4, IL-10, and IL-6, in NK cells. It was reported that IL-27 stimulated the transcription of IFN-ɣ mRNA in human NK cells exposed to IL-12 and a high concentration of IL-15 but not in those exposed to IL-27 alone [33]. Although the effect of IL-27 on T cells has been characterized [43, 44], its effect on human NK cells remains poorly defined. In this work, we investigated whether IL-27 induced IFN-ɣ secretion when it was added with IL-15 and lL-18 (Fig. 1f).

IL-27 notably stimulated NK cell cytotoxic activity (Fig. 1e) in this study. The NK cells stimulated with IL-15/IL-18/IL-27 showed significantly increased cytotoxicity in response to contact with K562 and A2780 as target cells compared to that mediated by other single cytokines or combinations, such as IL-2, IL-15, and IL-15/IL-18, and NK-92 cells. Interestingly, it was reported that IL-27 not only exerts direct activation on NK cells but also primes them for IL-18 responsiveness [31]. In accordance, we demonstrated here that IL-18 and IL-27 can act synergistically with IL-15 in stimulating in vitro proliferation, cytotoxicity activity, and IFN-ɣ secretion, although IL-18 and IL-27 were not sufficient to drive NK cell expansion. NK cells constitutively express IL-18 receptor alpha and are stimulated by IL-18 to produce IFN-ɣ [27].

Human NK cells can recognize cancer cells, resulting in phenotypic changes. Here, we identified that NK cells stimulated with IL-15/IL-18/IL-27 promoted the upregulation of NKp30 and NKp44 (Additional file 1: Table S2). A previous report mentioned that through the upregulation of NKp46, IL-27 could overcome resistance to NK cell-mediated cytotoxicity [45]; however, we did not observe that our cytokine combinations induced upregulation of NKp46.

Several papers have reported that IL-27 induces granzyme B and perforin production in CD8 T cells [46]. However, the effects of IL-27 on human NK cells remain poorly defined. To clear *aberrant* cells, such as cancer cells or infected cells, NK cells rapidly mobilize lytic granules, such as perforin and granzyme B, to the contact zone to initiate target cell lysis by caspase-dependent [47] and caspase-independent pathways [48]. In this study, we found that NK cells stimulated with IL-15/IL-18/IL-27 showed the highest cytotoxic activity compared to those stimulated with IL-15/IL-18, IL-2 alone, or IL-15 alone, and this effect was accompanied by increased intracytoplasmic perforin granule accumulation. In Fig. 3b, our data suggest that IL-27 acted synergistically with IL-15 and that IL-18 contributes to NK cell cytotoxic activation by increasing cytolytic granule, perforin, and granzyme accumulation (Additional file 2: Figure S5). We also demonstrated here that IL-15/IL-18/IL-27-stimulated NK cells retained high perforin expression and underwent some target-dependent degranulation and that target cancer cells experienced caspase-dependent apoptosis (Additional file 2: Figure S3).

## Conclusions

We demonstrated here for the first time that incubation of human primary NK cells with the cytokine combination of IL-15/IL-18/IL-27 enhanced proliferation and NK cell-mediated cytotoxic activity. This cytokine combination can also affect the production of IFN-ɣ, granzyme B and perforin, and this increased cytotoxicity was mediated by caspase-dependent apoptosis. Taken together, these data indicate that IL-27 can act as an important regulator in human NK cell proliferation and activation.

In addition, we suggest a combination of IL-15, IL-18, and IL-27 in human NK cell culture can lead to increased effectiveness of NK cell-mediated immunotherapeutics against cancers or infectious diseases.

## Additional files

Additional file 1: **Table S1.** Distribution of PBMCs and human NK cell number in healthy donors (*n* = 26). **Table S2.** Percentage of NK cells receptor positive cells in CD3-CD56+ primary NK cells from healthy donors (*n* = 9). **Table S3.** Percentage of NK cells receptor positive cells in CD3-CD56+ NK cells from healthy donors (*n* = 3). (DOCX 37 kb)

Additional file 2: **Figure S1.** Gating strategy for flow cytometry. **Figure S2.** Gating strategy to identify NK subsets. **Figure S3.** NK cytotoxicity against ovarian cancer cells. **Figure S4.** Detection of NK cell surface receptor expression in NK cell lines, NK-92, and NK-92MI. **Figure S5.** Western blotting analysis for perforin and granzyme B. **Figure S6.** Overview of human cytokine-mediated NK cell responses. (DOCX 1791 kb)

## Data Availability

All data generated and analyzed during this study are included within this published article and its supplementary information files.List of abbreviations.

## Abbreviations

CD: Cluster of differentiation
CTLA-4: Cytotoxic T lymphocyte-associated antigen 4
ELISA: Enzyme-linked immunosorbent assay
IFN-γ: Interferon gamma
IL: Interleukin
KIR: Killer cell immunoglobulin-like receptor
MHC: Major histocompatibility complex
NCRs: Natural cytotoxicity receptors
NK cells: Natural killer cells
NKG2D: Natural killer group 2D
PBMCs: Peripheral blood mononuclear cell
PBS: Phosphate buffered saline
PD-1: Programmed death-1
