# Correction to: IL-27 enhances IL-15/IL-18-mediated activation of human natural killer cells

**DOI:** 10.1186/s40425-019-0688-8

**Published:** 2019-08-08

**Authors:** Yeon Ho Choi, Eun Jin Lim, Se Wha Kim, Yong Wha Moon, Kyung Soon Park, Hee-Jung An

**Affiliations:** 1 Institute for Clinical Research, CHA Bundang Medical Center, CHA University, Sungnam, Gyeonggi-do Republic of Korea; 2 Department of Pathology, CHA Bundang Medical Center, CHA University, Sungnam, Gyeonggi-do Republic of Korea; 3 Department of Medical Oncology, CHA Bundang Medical Center, CHA University, Sungnam, Gyeonggi-do Republic of Korea; 4 Department of Biomedical Science, CHA University, Sungnam, Gyeonggi-do Republic of Korea

**Correction to: J Immuno Ther Cancer (2019) 7:168**

**https://doi.org/10.1186/s40425-019-0652-7**

After publication of the original article [1], the authors reported that three figures published in their manuscript are wrong.

The correct version of the figures can be found below:

Figs. 1, 2, 4

**Fig. 1:**
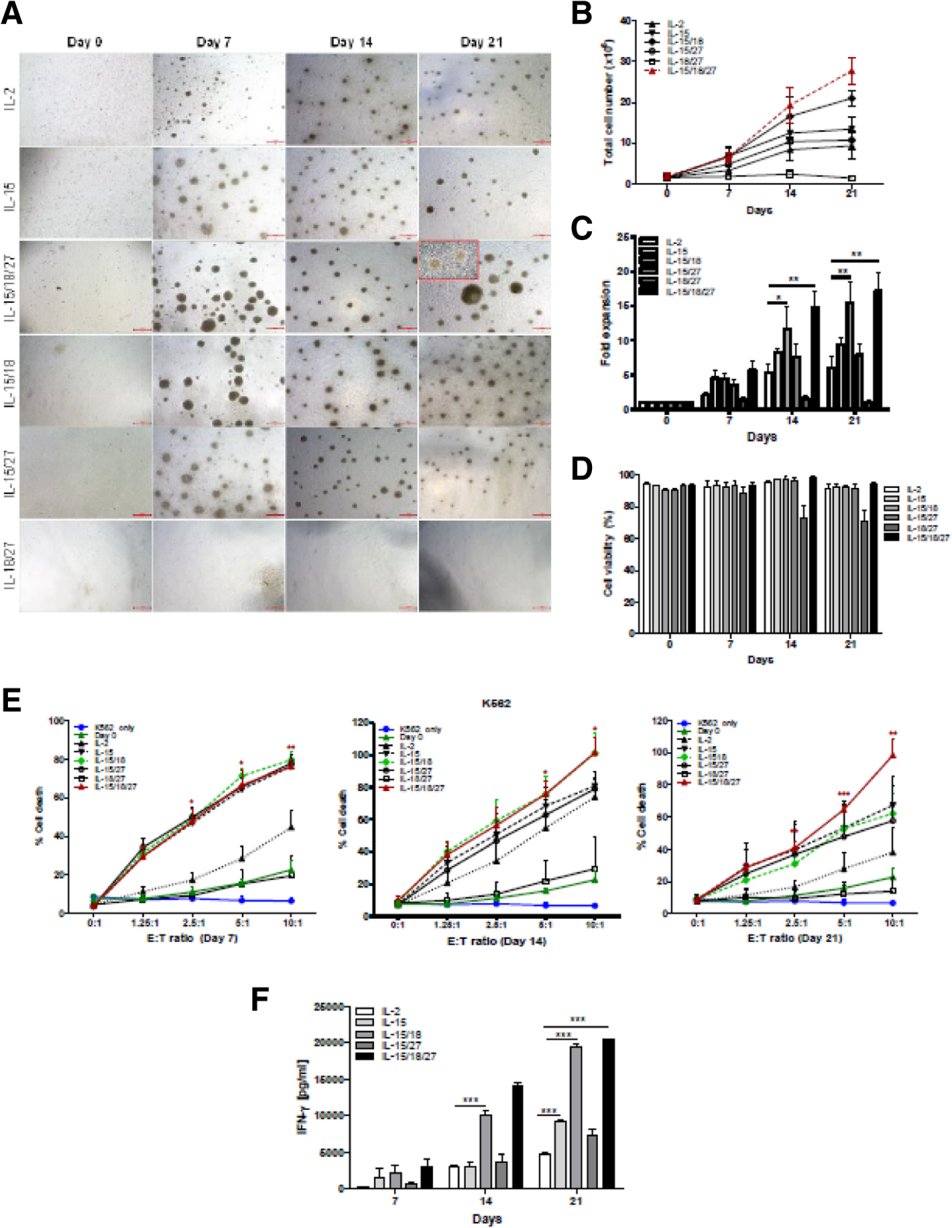
Cytokine regulation of the proliferation and cytotoxicity of primary NK cells*.* **a** CD3-CD56+ NK cells (0.6–1.8 × 10^6^ / well) isolated from PBMCs were cultured in the indicated cytokines for 21 days. Primary NK cells were imaged using an inverted microscope and counted. Representative images of aggregates of growing primary NK cells in different culture conditions. Bars represent 500 μm; original magnification × 40. **b** The graph represents the total NK cell number of each group. Symbols indicate cytokine treatment groups (*n* = 3 / group): IL-2 (▲), IL-15 (▼), IL-15/18 (◆), IL-15/27 (○), IL-18/27 (□), and IL-15/18/27 (*red*). **c**. Fold expansion of NK cell numbers compared with those on day 0 following culture of CD3-CD56+ NK cells with the indicated cytokines. The graphs show the mean ± SD. * *P* < 0.05, ** *P* < 0.01, and *** *P* < 0.001, compared with day 0. **d** NK cell viability. The viable cell numbers were determined by trypan blue staining on days 7, 14 and 21. * *P* < 0.05, compared with day 14. **e** NK cytotoxicity assays of various cytokine-stimulated NK cells with K562 target cells on days 7, 14 and 21. The E:T ratios ranged from 0:1 to 10:1. After 4 h of incubation at 37 °C, the lysis of target cells was measured by ELISA. E:T indicates the effector-to-target ratio. The cytolytic activity of human NK cells stimulated with IL-15/18/27 toward K562 cells was significantly increased (*P < 0.05, **P < 0.01, *** *P* < 0.001) compared with that of resting NK cells (day 0) at the same E:T ratio. **f** Supernatants were analyzed for IFN-ɣ secretion by ELISA. The data presented are the mean ± SD of three separate experiments., *** *P* < 0.001, compared with IL-2 treated group

**Fig. 2:**
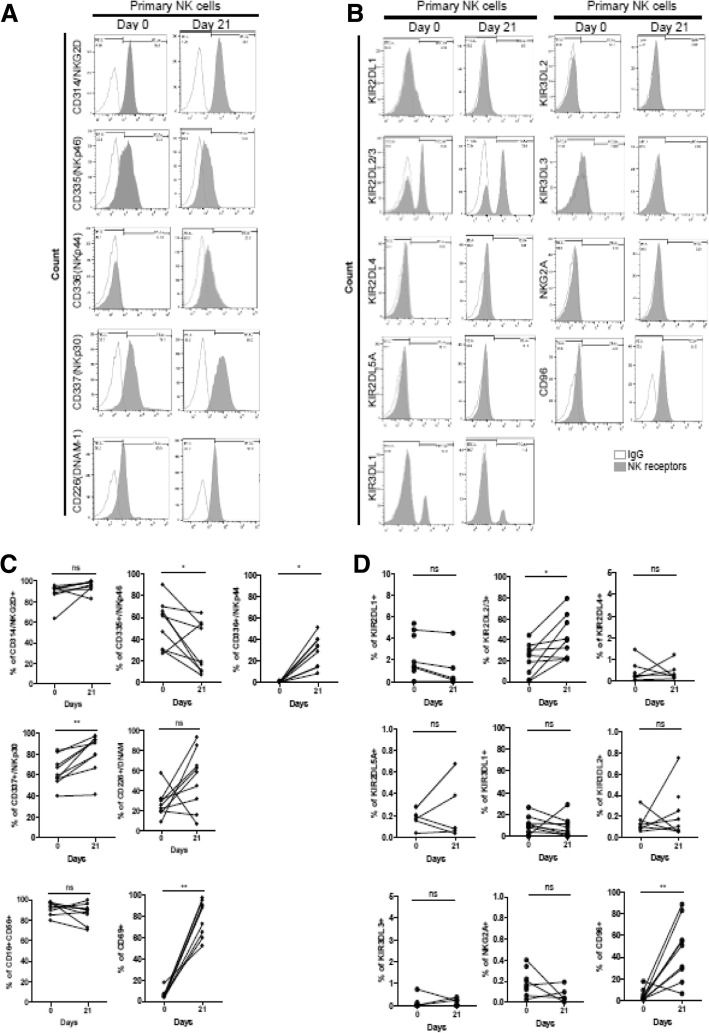
Flow cytometry analysis of NK cell receptors on human primary NK cells. (A) Cell surface expression of the indicated molecules on primary human NK cells on day 0 and day 21. Cells were stimulated with cytokine combinations (IL-15, IL-18, and IL-27) for 21 days. Primary NK cells from healthy donors were stained for expression of NK cell activating receptors (**a**) and inhibitory receptors (**b**), as indicated. Histograms show representative examples of NK cell receptor expression (shadow area) and show the percentage of NK cells positive for a given receptor relative to the isotype control (gray lines). NK cells were gated as viable, single, CD3-CD56+ cells. (**c**-**d**) Statistical analysis for the difference in NK cell receptor expression between day 0 and day 21. Significant differences are indicated in the graph as follows: *P < 0.05 and ** *P* < 0.01, compared with day 0. n.s: not significant

**Fig. 4:**
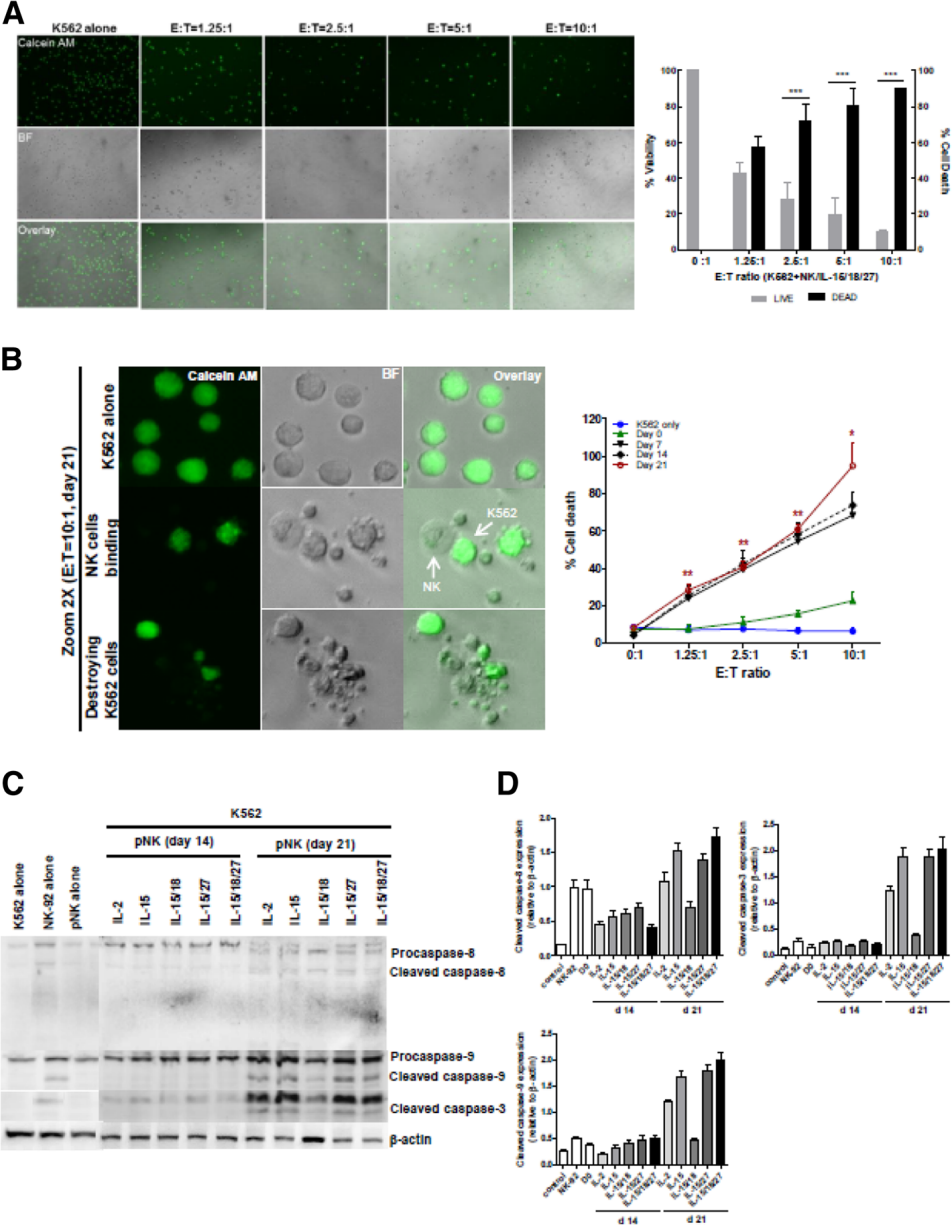
Measurement of NK cell cytotoxicity by imaging cytometry. **a** K562 target cells were stained with calcein AM. After 4 h of incubation with 21-day-expanded NK cells (stimulated with IL-15/18/27), fluorescence images show progressive loss of fluorescence intensity of the K562 cells at various E:T ratios. Representative bright field, calcein, and overlay images showing E:T ratio-dependent target cell killing. Original magnification × 100. The graph represents the percentage of viable or dead target cells. *** *P* < 0.001 was considered signigicant. **b** A high power view of the calcein AM assay showing the progress of NK cell killing. Nearly all of the target cells were killed in a 10:1 effector-to-target cell sample, while calcein AM-labeled K562 cells were not killed in the control image. Bright-field and fluorescence overlay images of calcein show K562 cells undergoing apoptotic death following interaction with NK cells. The images were derived from a Zeiss LSM 510 microscope (*left*). The graph (*right*) represents cytotoxicity against K562 cells with expanded NK cells on days 7, 14 and 21 over the 4 h of the assay. *P < 0.05, ** *P* < 0.01, ***P < 0.001 compared with day 0. Symbols indicate cytokine treatment groups (*n* = 3 / group): day 0 (▲), day 7 (▼), day 14 (◆), day 21 (red ○), and K562 cells only (●). **c** Immunoblot analysis for caspase-8, − 9 and − 3 activation. K562 cells were cocultured with primary NK cells for 4 h. Immunoblotting was performed with antibodies specific for caspase-8, − 9 and − 3 and their cleaved forms. β-actin was used as an internal standard. **d** Protein bands were quantitated by densitometric analysis. The ratio of the intensity of protein bands relative to that of β-actin was calculated. Bar graph represents the relative expression of cleaved caspase-8, − 9 and − 3 proteins. Experiments were repeated three times with similar results

The original article has been corrected.

The publisher apologies for the inconvenience.
