# Supplementary material for: IL-27 enhances IL-15/IL-18-mediated activation of human natural killer cells
Source: J Immunother Cancer. 2019 Jul 5;7:168. doi: 10.1186/s40425-019-0652-7 (PMC6612093; doi:10.1186/s40425-019-0652-7)
Supplement: Supplementary file 1 — Table S1. Distribution of PBMCs and human NK cell number in healthy donors (n = 26). Table S2. Percentage of NK cells receptor positive cells in CD3-CD56+ primary NK cells from healthy donors (n = 9). Table S3. Percentage of NK cells receptor positive cells in CD3-CD56+ NK cells from healthy donors (n = 3). (DOCX 37 kb) [file 40425_2019_652_MOESM1_ESM.docx]

**Table S1．Distribution of PBMCs and human NK cell number in healthy donors (n=26).**

| **Gender** | **Age (years)** | **No. of Healthy Control** | **Average (Mean ± SD^†^)** | | |
| --- | --- | --- | --- | --- | --- |
|  |  |  | **PBMC^*^ (X10^8)** | **NK (X10^7)** | **Yield (%)** |
| Femlae | 20-29 | 5 | 2.564 ± 0.952 | 2.526 ± 1.259 | 10.110 ± 3.770 |
|  | 30-39 | 5 | 1.634 ± 0.461 | 1.220 ± 0.139 | 7.880 ± 1.970 |
|  | 40-49 | 2 | 1.990 ± 1.294 | 2.460 ± 2.242 | 11.030 ± 4.100 |
|  | **Total** | **12** | 2.080 ± 0.871 | 1.970 ± 1.216 |  |
| Male | 20-29 | 2 | 2.385 ± 0.587 | 2.425 ± 0.955 | 9.977 ± 1.547 |
|  | 30-39 | 9 | 1.965 ± 0.579 | 2.294 ± 1.264 | 11.522 ± 4.171 |
|  | 40-49 | 3 | 1.237 ± 0.220 | 2.100 ± 0.383 | 17.681 ± 5.930 |
|  | **Total** | **14** | 1.869 ± 0.617 | 2.271 ± 1.043 |  |

*** Abbreviations: PBMCs; peripheral blood mononuclear cells, NK; Natural killer cells. ^†^SD; Standard deviation.

Additional file 1: **Table S1.** A total of twenty-six healthy donor participants were included for this study, and there were no significant differences in age and gender between healthy donors.

**Table S2．Percentage of NK cells receptor positive cells in CD3-CD56+ primary NK cells from healthy donors (n=9).**

| **HD #**  **(Day 0)** | **CD314**  **(NKG2D)** | **CD335**  **(NKp46)** | **CD336**  **(NKp44)** | **CD337**  **(NKp30)** | **CD226**  **(DNAM)** | **KIR2DL1**  **(CD158a)** | **KIL2DL2/3**  **(CD158b)** | **KIR2DL4**  **(CD158d)** | **KIR2DL5A**  **(CD158f1)** | **KIR3DL1**  **(CD158e1)** | **KIR3DL2**  **(CD158k)** | **KIR3DL3**  **(CD158z)** | **NKG2A** | **CD96** | **CD69** | **CD16** |
| --- | --- | --- | --- | --- | --- | --- | --- | --- | --- | --- | --- | --- | --- | --- | --- | --- |
| 27-0 | 92.2 | 28.9 | 0.13 | 39.7 | 18.3 | 0.011 | 0.66 | 0.18 | 0.17 | 0.044 | 0.086 | 0 | 0.16 | 2.78 | 4.35 | 97.5 |
| 26-0 | 92.8 | 26 | 0.077 | 57 | 8.36 | 1.01 | 34.7 | 0.21 | 0.28 | 11.3 | 0.077 | 0 | 0.14 | 2.06 | 4.94 | 96.6 |
| 24-0 | 95.6 | 29.7 | 0.11 | 53.7 | 19.4 | 1.3 | 44.2 | 0.21 | 0.15 | 8.44 | 0.16 | 0 | 0.062 | 1.71 | 3.56 | 95.7 |
| 23-0 | 63.4 | 65.3 | 0.54 | 83.4 | 57.3 | 1.42 | 18.8 | 1.44 | 1.16 | 9.73 | 0.33 | 0 | 0.4 | 17.6 | 17.3 | 79.4 |
| 22-0 | 87.1 | 46.2 | 0.096 | 59.5 | 21.8 | 1.82 | 26.9 | 0.27 | 0.19 | 11.8 | 0.099 | 0.73 | 0.027 | 0.94 | 6.25 | 93.3 |
| 21-0 | 88.8 | 60.9 | 1.77 | 69.6 | 31.5 | 5.37 | 1.45 | 0.013 | 0.2 | 1.23 | 1.01 | 0 | 0.12 | 1.57 | 3.64 | 89.3 |
| 20-0 | 93.5 | 69.9 | 0.27 | 81.5 | 31.3 | 4.26 | 30.4 | 0.69 | 5.71 | 17.9 | 0.12 | 0 | 0.2 | 5 | 4.65 | 91.9 |
| 19-0 | 93.1 | 62.6 | 0.26 | 56.6 | 29.4 | 4.79 | 8.92 | 0.057 | 0.034 | 4.26 | 0.057 | 0 | 0.22 | 1.59 | 3.65 | 96.7 |
| 18-0 | 92.6 | 90 | 0 | 66.7 | 25.2 | 10.4 | 24.9 | 0.36 | 0.27 | 26.6 | 0.12 | 0.12 | 0.34 | 9.32 | 6.91 | 85 |
| **Mean (%)** | 88.31 | 48.69 | 0.41 | 62.63 | 27.17 | 2.50 | 20.75 | 0.38 | 0.99 | 8.09 | 0.24 | 0.09 | 0.17 | 4.16 | 6.04 | 92.550 |
| **SD** | 10.42 | 18.29 | 0.57 | 14.73 | 14.49 | 2.00 | 16.01 | 0.47 | 1.94 | 5.97 | 0.32 | 0.26 | 0.11 | 5.57 | 4.64 | 6.006 |
| **HD #**  **(Day 21)** | **CD314**  **(NKG2D)** | **CD335**  **(NKp46)** | **CD336**  **(NKp44)** | **CD337**  **(NKp30)** | **CD226**  **(DNAM)** | **KIR2DL1**  **(CD158a)** | **KIL2DL2/3**  **(CD158b)** | **KIR2DL4**  **(CD158d)** | **KIR2DL5A**  **(CD158f1)** | **KIR3DL1**  **(CD158e1)** | **KIR3DL2**  **(CD158k)** | **KIR3DL3**  **(CD158z)** | **NKG2A** | **CD96** | **CD69** | **CD16** |
| 27-21 | 99.5 | 6.13 | 39.9 | 41 | 58.1 | 0 | 22.5 | 0.51 | 0.38 | 0 | 0.25 | 0 | 0.19 | 55.7 | 87.9 | 72.4 |
| 26-21 | 99.7 | 54.2 | 28.7 | 95.8 | 85 | 16.8 | 40.9 | 9 | 3.15 | 1.7 | 1.09 | 0.24 | 3.4 | 83 | 97.3 | 89.9 |
| 24-21 | 98.6 | 18.4 | 14.4 | 79.2 | 15.3 | 0.16 | 79.5 | 1.2 | 0.032 | 0.44 | 0.053 | 0 | 0.039 | 54.7 | 72.6 | 85.4 |
| 23-21 | 93.99 | 8.93 | 40.1 | 90 | 6.21 | 0.31 | 21.4 | 0.21 | 0.08 | 4.76 | 0.05 | 0 | 0.03 | 6.32 | 52.0 | 70.2 |
| 22-21 | 96.5 | 10.2 | 14.8 | 78.6 | 31.4 | 1.26 | 64.1 | 0.29 | 0.075 | 11.5 | 0.065 | 0.21 | 0.095 | 31.1 | 59.6 | 91.2 |
| 21-21 | 95.1 | 49.5 | 33.3 | 92.6 | 93.1 | 25.4 | 39.7 | 19.6 | 1.33 | 29.1 | 0.38 | 0.37 | 2.02 | 82.5 | 90.1 | 99.4 |
| 20-21 | 99.1 | 63.8 | 51.2 | 97.1 | 44.4 | 21.2 | 32.3 | 0.21 | 4.56 | 8.42 | 0.17 | 0 | 0 | 50.8 | 90.4 | 95.7 |
| 19-21 | 82.6 | 15.9 | 8.37 | 66.4 | 64.6 | 4.48 | 56.4 | 0.12 | 0.054 | 2.37 | 0.1 | 0 | 3.21 | 16.9 | 64.9 | 89.9 |
| 18-21 | 91.8 | 52.8 | 34.1 | 93 | 62 | 12.6 | 22.4 | 14.5 | 0.67 | 13.7 | 0.75 | 5.07 | 1.16 | 88.6 | 95.1 | 88 |
| **Mean (%)** | 95.64 | 28.38 | 28.85 | 80.09 | 49.76 | 8.70 | 44.60 | 3.89 | 1.21 | 7.29 | 0.27 | 0.10 | 1.12 | 47.63 | 76.85 | 86.76 |
| **SD** | 5.68 | 23.38 | 15.09 | 18.93 | 31.30 | 10.64 | 20.54 | 7.03 | 1.73 | 9.69 | 0.35 | 0.15 | 1.51 | 28.07 | 16.80 | 10.43 |

Additional file 1: **Table S2.** Expression of NK cell receptor was measured by flow cytometry. NK cells stimulated with cytokine combination of IL-15/18/27.

**Table S3．Percentage of NK cells receptor positive cells in CD3-CD56+ NK cells from healthy donors (n=3).**

| **Target gene** | **Day 0** | | **Day 21** | | | | | |
| --- | --- | --- | --- | --- | --- | --- | --- | --- |
|  |  |  | **IL-2^Hi^** | | **IL-2** | | **IL-15** | |
|  | **Mean** | **SD** | **Mean** | **SD** | **Mean** | **SD** | **Mean** | **SD** |
| **CD314 (NKG2D)** | 85.350 | 2.051 | 54.20 | 3.458 | 31.20 | 13.011 | 61.70 | 25.03 |
| **CD335 (NKp46)** | 24.700 | 4.808 | 7.70 | 3.090 | 6.31 | 3.267 | 9.19 | 1.15 |
| **CD336 (NKp44)** | 0.139 | 0.143 | 7.93 | 2.751 | 12.30 | 3.253 | 22.80 | 1.10 |
| **CD337 (NKp30)** | 47.400 | 15.274 | 26.50 | 5.445 | 46.60 | 4.844 | 59.50 | 0.71 |
| **CD226 (DNAM)** | 14.870 | 17.013 | 8.54 | 1.457 | 30.90 | 13.117 | 5.22 | 0.54 |
| **KIR2DL1 (CD158a)** | 0.141 | 0.197 | 0.70 | 0.905 | 0.78 | 0.003 | 0.27 | 0.04 |
| **KIL2DL2/3 (CD158b)** | 5.023 | 7.039 | 82.50 | 9.899 | 79.90 | 1.549 | 0.41 | 0.30 |
| **KIR2DL4 (CD158d)** | 0.109 | 0.073 | 0.08 | 0.417 | 0.25 | 0.212 | 2.89 | 1.27 |
| **KIR2DL5A (CD158f1)** | 0.199 | 0.228 | 0.17 | 0.035 | 0.13 | 0.023 | 0.39 | 0.23 |
| **KIR3DL1 (CD158e1)** | 3.040 | 4.299 | 0.72 | 0.643 | 1.11 | 0.002 | 0.33 | 0.13 |
| **KIR3DL2 (CD158k)** | 0.385 | 0.205 | 0.09 | 0.110 | 0.10 | 0.009 | 0.59 | 0.57 |
| **KIR3DL3 (CD158z)** | 0.000 | 0.000 | 0.17 | 0.007 | 4.58 | 0.948 | 1.53 | 0.81 |
| **NKG2A** | 0.134 | 0.136 | 0.00 | 0.000 | 0.07 | 0.065 | 0.27 | 0.24 |
| **CD96** | 5.020 | 5.629 | 78.50 | 27.577 | 66.60 | 32.527 | 44.50 | 34.63 |
| **CD69** | 2.685 | 0.346 | 7.26 | 1.697 | 16.10 | 6.930 | 44.00 | 41.01 |
| **CD16** | 89.250 | 0.071 | 44.50 | 9.687 | 50.30 | 13.789 | 69.10 | 29.56 |

| **Target gene** | **Day 0** | | **Day 21** | | | | | | | |
| --- | --- | --- | --- | --- | --- | --- | --- | --- | --- | --- |
|  |  |  | **IL-2^Hi^** | | **IL-2** | | **IL-15** | | **IL-15/18/27** | |
|  | **Mean** | **SD** | **Mean** | **SD** | **Mean** | **SD** | **Mean** | **SD** | **Mean** | **SD** |
| **PD-1** | 4.517 | 0.726 | 0.27 | 0.225 | 0.6 | 0.008 | 0.43 | 0.59 | 0.71 | 0.3 |
| **CTLA-4** | 0.797 | 0.396 | 0.18 | 0.057 | 0.35 | 0.346 | 0.75 | 1.05 | 0.41 | 0.07 |

Additional file 1: **Table S3.** Expression of NK cell receptor was measured by flow cytometry. NK cells stimulated with high dose IL-2, IL-2, and IL-15. We measured PD-1 and CTLA-4 expression in each group. There were no significant differences between high dose IL-2, IL-2, IL-15, and IL-15/18/27.
