# Supplementary material for: IL-27 enhances IL-15/IL-18-mediated activation of human natural killer cells
Source: J Immunother Cancer. 2019 Jul 5;7:168. doi: 10.1186/s40425-019-0652-7 (PMC6612093; doi:10.1186/s40425-019-0652-7)
Supplement: Supplementary file 2 — Figure S1. Gating strategy for flow cytometry. Figure S2. Gating strategy to identify NK subsets. Figure S3. NK cytotoxicity against ovarian cancer cells. Figure S4. Detection of NK cell surface receptor expression in NK cell lines, NK-92, and NK-92MI. Figure S5. Western blotting analysis for perforin and granzyme B. Figure S6. Overview of human cytokine-mediated NK cell responses. (DOCX 1791 kb) [file 40425_2019_652_MOESM2_ESM.docx]

**Figures　S1**

**A**

**PBMC**

**Before culture**

**After culture**

**B**

**Figure S1.** Gating strategy for flow cytometry. (A) The gating strategy used to identify the different immune cell populations. Isolated PBMCs (*upper panel*) and NK cells (before culture; *middle panel,* after 21 days of culture; *lower panel*) from blood and lymphocytes were first gated based on forward scatter and side scatter. T cells were then identified based on CD3 positivity, and NK cells were identified based on the CD3-CD56+ profile. From the T cell, CD3+ gate, CD14+ monocytes, and CD19+ B cells were delineated. The figure refers to the frequency in a corresponding gate or quadrant. (B) The percentages of CD3−CD56+, CD56+CD16+, CD3+, CD14+, and CD19+ cells were analyzed by flow cytometric analyses (n = 9). Representative FACS dot plots are presented.

**Figure S2**

**A**

**B**

| **NK subsets** | **IL-15/18/27** | | **IL-2^Hi^** | **IL-2** | **IL-15** |
| --- | --- | --- | --- | --- | --- |
|  | **d0** | **d21** | **d21** | **d21** | **d21** |
| CD56^bright^CD16- | 2.80 ± 0.63 | 3.24 ± 3.07 | 2.11 ± 0.13 | 2.46 ± 0.45 | 5.04 ± 3.85 |
| CD56^dim^ CD16+ | 90.8 ± 1.00 | 93.1 ± 3.38 | 90.3 ± 3.63 | 93.5 ± 5.60 | 88.7 ± 4.23 |
| CD56^neg^ CD16+ | 0.23 ± 0.09 | 0.57 ± 0.58 | 3.06 ± 2.63 | 2.60 ± 0.89 | 1.62 ± 0.75 |

**Figure S2.** Gating strategy to identify NK subsets. (A) Flow cytometry dot plot of CD56 versus CD16 after gating on viable, single, CD3− NK cells from peripheral blood mononuclear cells (PBMCs) of a healthy donor. In panels that included CD16 as a marker, NK cell subsets were identified by sequentially gating on lymphocytes, then CD3- cells, and then cells expressing CD16 and/or CD56. NK cell subsets were identified as CD56^bright^ (upper right gate), CD56+CD16+, and CD56-CD16+ (lower right gate). In panels without CD16 staining, NK cells were identified as CD3- lymphocytes that were either bright (CD56^bright^) or dim (CD56^dim^) for by CD56 staining; the CD56- population could not be distinguished. (B) The proportion of NK subsets before and after cultures with various cytokines. Percentages of the different natural killer (NK) cell subsets relative to the total NK cell population (100%) from PBMCs of a series of healthy donors. Treatment groups ; IL-15/18/27 (n=9), high dose IL-2, IL-2, and IL-15 (n=3).

**Figures　S3**

**A**

**B**

**C**

**Figure S3.** NK cytotoxicity against ovarian cancer cells. (A) NK cytotoxicity assays of various cytokine-stimulated NK cells with A2780 target cells on days 7, 14 and 21. The E: T ratios ranged from 0:1 to 10:1. After 4 hours of incubation at 37 °C, the lysis of target cells was measured by ELISA. E: T indicates the effector-to-target ratio. The cytolytic activity of human NK cells with IL-15/18/27 stimulation toward A2780 cells was significantly increased (**P* < 0.05, ***P* < 0.01, compared with day 0) compared with that of resting NK cells (day 0) at the same E: T ratio. Symbols indicate cytokine treatment groups (n = 3 / group). (B) Immunoblot analysis for caspase-8, -9 and -3 activation. A2780 cells were cocultured with a combination of IL-15-, IL-18-, and IL-27-stimulated NK cells for 4 hours. Immunoblotting was performed with antibodies specific for caspase-8, -9 and -3 and their cleaved forms. β-actin was used as an internal standard. (C) Protein bands were quantitated by densitometric analysis. The ratio of the intensity of protein bands relative to that of β-actin was calculated. The bar graph represents the relative expression of cleaved caspase-8, -3 and -9 proteins. Experiments were repeated three times with similar results.

**Figures　S4**

**Ａ**

**Ｂ**

| **Target** | **NK92MI (n=3)** | | **NK92 (n=3)** | |
| --- | --- | --- | --- | --- |
|  | **Mean (%) ± SD** | | **Mean (%) ± SD** | |
| **CD314 (NKG2D)** | 79.60 | 18.01 | 50.17 | 7.21 |
| **CD335 (NKp46)** | 4.17 | 3.75 | 1.38 | 1.81 |
| **CD336 (NKp44)** | 27.27 | 3.69 | 10.38 | 13.04 |
| **CD337 (NKp30)** | 83.30 | 18.68 | 87.13 | 9.24 |
| **CD226 (DNAM)** | 3.58 | 1.53 | 1.41 | 2.04 |
| **KIR2DL1 (CD158a)** | 0.09 | 0.09 | 0.02 | 0.02 |
| **KIL2DL2/3 (CD158b)** | 0.13 | 0.02 | 0.15 | 0.05 |
| **KIR2DL4 (CD158d)** | 0.14 | 0.15 | 1.38 | 1.26 |
| **KIR2DL5A (CD158f1)** | 0.60 | 0.89 | 0.59 | 0.49 |
| **KIR3DL1 (CD158e1)** | 3.25 | 4.93 | 0.03 | 0.02 |
| **KIR3DL2 (CD158k)** | 0.14 | 0.06 | 0.10 | 0.17 |
| **KIR3DL3 (CD158z)** | 0.79 | 0.69 | 0.41 | 0.55 |
| **NKG2A** | 0.04 | 0.02 | 0.20 | 0.20 |
| **CD96** | 84.80 | 13.02 | 62.33 | 52.42 |
| **CD69** | 14.02 | 5.14 | 2.16 | 3.67 |
| **CD16** | 1.80 | 0.72 | 23.60 | 37.60 |

**Figure S4.** Detection of NK cell surface receptor expression in NK cell lines, NK-92 and NK-92MI. (A) Representative bar graphs of expression of NK cell activating receptors (left panel) and inhibitory receptors (right panel) in the NK-92 and NK-92 MI cell lines. (B) The table shows the percentage of positive cells with NK surface receptors (mean ± SD). Data were pooled from three independent experiments.

**Figures　S5**

**
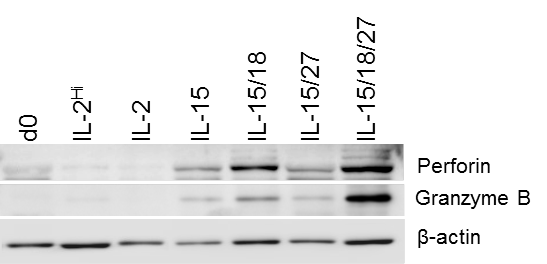
A**

**B**

**Figure S5.** Western blotting analysis for perforin and granzyme B. (A) CD3-CD56+ NK cells were stimulated with cytokines, namely, IL-2^hi^, IL-2 only, IL-15 only, IL-15/IL-18, IL-15/IL-27, and IL-15/IL-18/IL-27, for 21 days. The graph represents the relative expression of each protein. Protein expression was quantitated by densitometric analysis. The ratio of the intensity of protein bands relative to that of β-actin was calculated. Experiments were repeated three times with similar results. *P < 0.05, compared with cells cultured on day 0.

**Figures　S6**

**A**

**B**

**Figure S6.** Overview of human cytokine-mediated NK cell responses. (A) Cell culture procedure for NK cells derived from PBMCs. Flow diagram of the preparation of CD3-CD56+ NK cells following protocols (Ficoll-Hypaque density gradient centrifugation). CD3-CD56+ NK cells were isolated from the peripheral blood of healthy donors. NK cells were continuously cultured with various cytokines for 21 days. When the cells reached confluence, they were transferred to T25 flasks, T75 flasks, or T175 flasks. (B) Summary illustration of the effect of cytokine combinations on controlling NK cell responses. Resting NK cells increased in cell size and number when stimulated by various cytokines. In addition, the expression of perforin and granzyme B and secretion of IFN-γ, which are involved in cytotoxicity, also increased in NK cells stimulated with a combination of IL-15, IL-18, and IL-27. NK cells exerted their killing activity on targets by the production of perforin and granzyme B granules. Last, perforin and granzymes were released from those granules to induce death of target cells by apoptosis. Our data show that IL-27 enhanced IFN-γ, perforin, and granzyme B production by NK cells when paired with IL-15 and IL-18. IL-27 acted synergistically with IL-15 and IL-18 in NK cells.
